# Perceived technology usefulness for caregiving among unpaid caregivers: a National Cross-Sectional Study

**DOI:** 10.3389/fpubh.2025.1578701

**Published:** 2025-05-14

**Authors:** Matthew Lee Smith, Shinduk Lee, Malinee Neelamegam, Deborah Vollmer Dahlke, Jodi L. Southerland, Zachary G. Baker, Kris Pui Kwan Ma, Darina V. Petrovsky, Zahra Rahemi, Justine S. Sefcik, Juanita-Dawne R. Bacsu, Chung Lin Kew, Marcia G. Ory

**Affiliations:** ^1^Department of Health Behavior, School of Public Health, Texas A&M University, College Station, TX, United States; ^2^Center for Community Health and Aging, Texas A&M University, College Station, TX, United States; ^3^College of Nursing, The University of Utah, Salt Lake City, UT, United States; ^4^Department of Biostatistics and Epidemiology, School of Public Health, University of North Texas Health Science Center, Fort Worth, TX, United States; ^5^DVD Associates LLC, Austin, TX, United States; ^6^Department of Community and Behavioral Health, East Tennessee State University, Johnson City, TN, United States; ^7^Edson College of Nursing and Health Innovation, Arizona State University, Tempe, AZ, United States; ^8^Department of Family Medicine, University of Washington, Seattle, WA, United States; ^9^School of Nursing, Duke University, Durham, NC, United States; ^10^School of Nursing, Clemson University, Clemson, SC, United States; ^11^College of Nursing and Health Professions, Drexel University, Philadelphia, PA, United States; ^12^School of Nursing, Thompson Rivers University, Kamloops, BC, Canada; ^13^Department of Environmental and Occupational Health, School of Public Health, Texas A&M University, College Station, TX, United States

**Keywords:** unpaid caregiving, technology, perceived usefulness, older adults, paid caregiving

## Abstract

**Background:**

Technological advancements have the potential to improve caregiving quality and alleviate caregiver burden by providing tools for real-time communication, monitoring, and care coordination. To assist with technology adoption among the 53 million unpaid caregivers nationwide, efforts are needed to better understand caregivers’ perceptions about the usefulness of certain technologies for caregiving.

**Methods:**

Data were analyzed from a national sample of 483 unpaid caregivers using an internet-delivered questionnaire. All unpaid caregivers were eligible if they provided at least 8 h of weekly care for a care recipient aged 50 years or older. The primary dependent variable was the Perceived Technology Usefulness for Caregiving (PTUC) Scale, which is a composite score of six items ranging from 0 to 100. PTUC item responses were summed and averaged, and the overall PTUC scores were transformed into statistical tertiles (higher scores indicating more perceived technology usefulness for caregiving). An ordinal regression model was fitted to identify factors associated with higher PTUC tertiles.

**Results:**

Across tertiles, unpaid caregivers who were younger (Beta = −0.018, *p* = 0.030) and male (Beta = 0.422, *p* = 0.048) reported higher PTUC Scale scores. Compared to non-Hispanic white caregivers, Hispanic/Latino (Beta = 0.779, *p* = 0.010), African American (Beta = 1.064, *p* < 0.001), and Asian (Beta = 0.958, p = 0.010) caregivers reported higher PTUC Scale scores. Unpaid caregivers with lower financial insecurity (Beta = −0.010, *p* = 0.003), higher caregiver strain (Beta = 0.149, p < 0.001), and more satisfaction with the support they receive for caregiving (Beta = 0.009, *p* = 0.002) reported higher PTUC Scale scores. Unpaid caregivers whose care recipients had less cognitive impairment reported higher PTUC Scale scores (Beta = −0.245, *p* = 0.048).

**Conclusion:**

Findings indicate caregiver characteristics, caregiving dynamics, and available resources (financial and caregiving support) are associated with perceptions about the usefulness of technology for caregiving. The utility of technology for caregiving may be higher among unpaid caregivers with more caregiver strain or positive experiences with caregiving support.

## 1 Introduction

There are an estimated 53 million unpaid caregivers in the United States who assist their family members, friends, and neighbors to meet their household, health, and psychosocial needs (1). Unpaid caregivers frequently provide assistance in running errands, attending appointments, and performing activities of daily living. Caregiving tasks are driven by the needs and demands of the care recipient, which can be complicated when care recipients have mobility, sensory, and/or cognitive impairments (2, 3). Further, based on the demands of their care recipients, unpaid caregivers are susceptible to high levels of burden (4) and poor physical and mental health consequences (5, 6). Persisting high levels of caregiver burden are associated with increased depression and anxiety symptoms and greater susceptibility to cardiovascular diseases and hypertension (7). Worse caregiver mental health also predicts greater mortality among persons with dementia, even when accounting for care recipients’ age and disease severity (8).

In recent years, there has been a proliferation in technological solutions targeting caregivers (3). These technological advancements are diverse in format and function and can be available in the form of smartphone applications, digital platforms, and wearables (9). Evidence suggests that technology has potential to improve caregiving quality and alleviate caregiving strain or burden by providing tools for real-time communication, monitoring, and care coordination (10). Assistive technology may have mixed benefits for caregivers, impacting emotional, financial, and time-related strain differently. Caregivers have reported that technology can both reduce and increase caregiver-related burden (11, 12). For example, a technology may be used to alleviate worry and anxiety among long-distance caregivers rather than to optimize time spent providing care (10). Studies also show that technology can provide reminder systems to support medication management and activities of daily living; therapeutic activities such as cognitive games, relaxation exercises, and music-therapy; and self-management programs for caregivers to help deal with behavioral change in people with dementia (13–15). Moreover, research suggests that technology can provide more independence for people with cognitive impairment by enhancing social interaction and reducing boredom to support family interactions for the care recipient and unpaid caregiver, especially during extended periods of social isolation (16, 17).

To overcome potential barriers to technology use and assist with technology adoption among unpaid caregivers, efforts are needed to better understand caregivers’ perceptions about the usefulness of technology for caregiving. In this context, the purposes of this study were to identify: (a) the degree to which unpaid caregivers perceive technology to be useful for caregiving; and (b) factors associated with higher perceived technology usefulness for caregiving.

## 2 Methods

### 2.1 Participants and procedures

Data were collected using a cross-sectional, internet-delivered survey. Participants were recruited from a Qualtrics panel in November 2019. The eligibility criteria to participate in the study required that participants be ages 18 years or older and be a paid or unpaid caregiver of at least one non-institutionalized adult ages 50 years or older. Quota sampling was used to ensure the study sample was diverse in terms of geographic regions across the United States (Northwest, Midwest, West, and South), sex, age, ethnicity, and race. Additional information about the survey and sampling methods are reported elsewhere (18, 19). A total of 626 paid and unpaid caregivers completed the survey; 143 paid caregivers were omitted from analyses given this study’s focus on unpaid caregivers. The resulting analytic sample was 483 unpaid caregivers of non-institutionalized adults ages 50 years and older. The study was approved by the Texas A&M University Institutional Review Board (IRB2019-1128 M).

### 2.2 Measures

#### 2.2.1 Dependent variable

The dependent variable used in this study was the Perceived Technology Usefulness for Caregiving (PTUC) Scale, which was created by study investigators and assessed by summing the responses of six items. PTUC items measured caregivers’ perceptions that technology is useful for: (a) easing caregiving burdens; (b) enabling care recipients to live more independently; (c) enabling better quality of life for care recipients; (d) improving relationships with care recipients; (e) communicating with care recipients’ family and friends; and (f) communicating with care recipients’ healthcare team. Participants rated each item from 0 (not at all) to 100 (a great deal) to indicate the extent to which they perceived technology was useful for caregiving. Items were then summed and divided by six to calculate an average score for the six PTUC items (ranging from 0 to 100%), with higher scores indicating higher perceived usefulness of technology for caregiving. In the current sample of unpaid caregivers, the 6-item PTUC Scale had a single-factor solution using exploratory factor analysis and a Cronbach alpha of 0.924. Because of the positively skewed frequency distribution, statistical tertiles were used to operationalize the PTUC Scale into lowest [range from 0 to 47.17; mean = 28.87; standard deviation (SD) = 13.59], medium (range from 47.50 to 70.33; mean = 58.82; SD = 6.72), and highest (range from 70.50 to 100; mean = 84.80; SD = 9.41) levels, which were used in ordinal regression analyses.

#### 2.2.2 Care recipient characteristics and caregiving situation

Caregivers were asked to report information about their care recipient and caregiving situation. Caregivers indicated their care recipients’ age, cognitive status (i.e., healthcare provider ever told that the care recipient has mild cognitive impairment/memory problems or Alzheimer’s Disease/Dementia). Caregivers also reported if they lived with their care recipient (i.e., no, yes) and the number of weekly hours they provided care. Caregivers were also asked to rate their satisfaction with the help in caregiving they received from friends, family member, or neighbors in the past month (range from 0 to 100, with higher score indicating greater satisfaction).

#### 2.2.3 Social engagement activities

Caregivers were asked, “in the past 2 weeks, have you participated in in-person organizational gatherings such as: (a) social clubs, resident groups, or committees; (b) community organizations; (c) hobby or interest group organizations; (d) religious group meetings; and (e) coffee or meals with friends.” Response choices for each item were “no” (scored 0) and “yes” (scored 1) and summed to create a count variable ranging from 0 to 5. Higher scores for this count variable indicate more social engagement.

#### 2.2.4 Caregiving strain

The Caregiving Strain Scale is a composite score of eight items to assess ways in which caregiving has caused strain in their lives. This scale is a modification of a chronic disease strain scale (20, 21), which the authors tailored to be related to caregiving. Caregivers were asked, “as a result of your caregiving in the past 3 months, have the following situations occurred? (a) had strained family relationships; (b) had strained financial situation; (c) had to reduce social activities; (d) had to cut back on helping family/friends; (e) had to miss work; (f) had to reduce time volunteering in other ways; (g) reduced your usual amounts of exercise; and (h) reduced time on hobbies.” Higher scores indicate more caregiving-related strain. In the current sample of unpaid caregivers, the 8-item Caregiver Strain Scale had a single-factor solution using exploratory factor analysis and a Cronbach alpha of 0.809.

#### 2.2.5 Caregiver sociodemographic characteristics

Caregiver’s sociodemographic characteristics included age, sex (i.e., female, male), ethnicity (i.e., non-Hispanic/Latino, Hispanic/Latino), race (i.e., White, Black or African American, Asian or Pacific Islander, or Other or Multiple Races), education level (i.e., high school or less, some college or associates degree, college graduate or more), and employment status (i.e., not employed, employed). As a proxy to financial insecurity, participants were asked to report how their “finances usually work out at the end of the month” (i.e., some money left over, just enough money to make ends meet, not enough money to make ends meet), and they also reported their residential rurality (i.e., large metro area, medium metro area, small metro area, urban cluster, small town, or rural area).

### 2.3 Statistical analyses

All statistical analysis were performed using SPSS version 29.^1^ Frequencies and descriptive statistics were generated for all variables of interest and compared across PTUC Scale tertiles. Chi-square tests were used to identify distribution differences across PTUC Scale tertiles for categorical variables. One-way ANOVAs were used to assess the mean differences for continuous and count variables across PTUC Scale tertiles. An ordinal regression model was fitted to assess factors associated with increasing levels of PTUC Scale scores among unpaid caregivers.

## 3 Results

**Figure 1:**
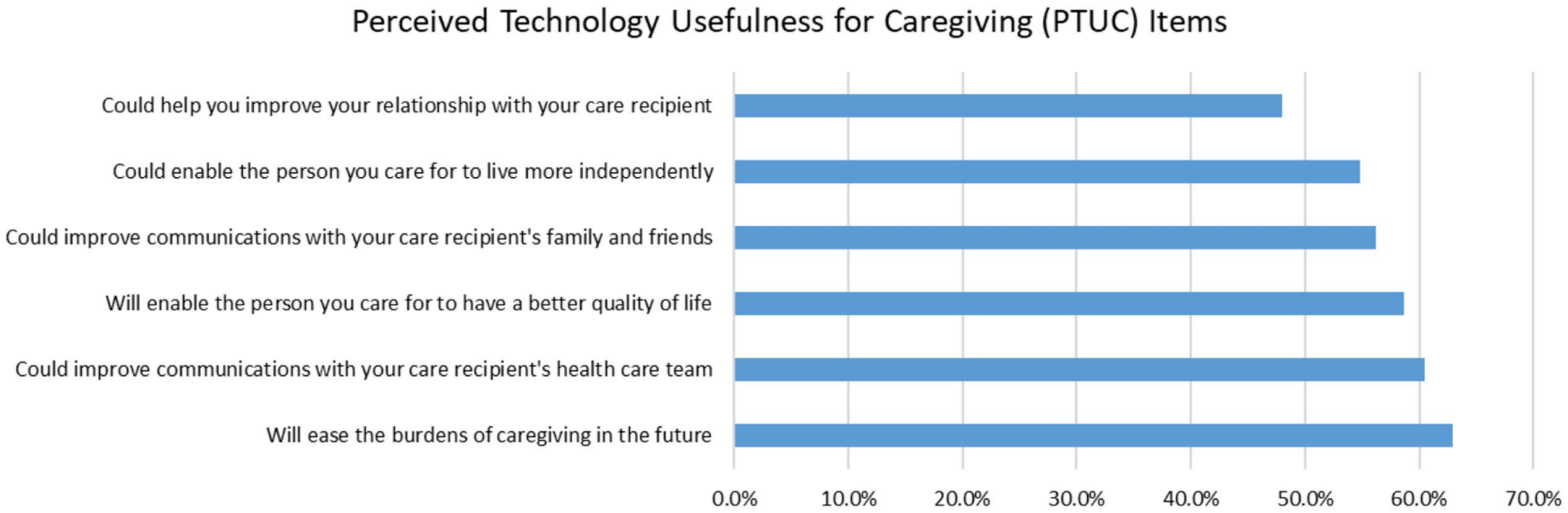
Perceived technology usefulness for caregiving (PTUC) items.

A total of 483 unpaid caregivers were included in this study. On average, unpaid caregivers had a PTUC Scale score of 56.83 (SD = 25.85). In terms of the individual PTUC Scale items (see Figure 1), unpaid caregivers perceived technology to be most useful to ease caregiving burdens in the future (62.9%), followed by to improve their communication with the care recipients’ health care team (60.5%), to enable the care recipient to have a better quality of life (58.7%), and to improve their communication with the care recipients’ family and friends (56.2%).

As shown in Table 1, the average age of these caregivers was 60.75 (SD = 12.13) years. Most caregivers were female (74.7%), non-Hispanic/Latino (90.1%), White (74.1%), and not employed (66.3%). Approximately 21% of caregivers had a high school education or less, 36.0% had some college or an associate’s degree, and 43.3% had a college degree or graduate-level education. The average age of care recipients was 74.86 (SD = 11.64) years. About 61% of the care recipients did not have cognitive impairment, 22.4% had mild cognitive impairment, and 16.6% had Alzheimer’s Disease or dementia. About 2-in-3 caregivers (64.6%) lived with their care recipient. On average, caregivers provided 55.40 (SD = 51.97) hours of care to their care recipients each week.

**Table 1:** Sample characteristics by PTUC Scale tertiles.

Variables	Total (*n* = 483)	Lowest (*n* = 161)	Middle (*n* = 161)	Highest (*n* = 161)	χ^2^ or f	*P*
Age	60.75 (±12.13)	63.27 (±11.62)	60.20 (±11.16)	58.78 (±13.15)	5.90	0.003
Sex					1.73	0.420
Female	74.7%	78.3%	73.9%	72.0%		
Male	25.3%	21.7%	26.1%	28.0%		
Hispanic					5.00	0.082
No	90.1%	93.8%	90.1%	86.3%		
Yes	9.9%	6.2%	9.9%	13.7%		
Race					30.39	<0.001
White	74.1%	86.3%	75.2%	60.9%		
Black or African American	14.1%	5.0%	14.3%	23.0%		
Asian or Pacific Islander	6.6%	4.3%	5.6%	9.9%		
Other or multiple races	5.2%	4.3%	5.0%	6.2%		
Education level					4.28	0.369
High school or less	20.7%	21.7%	22.4%	18.0%		
Some college/associates	36.0%	32.9%	40.4%	34.8%		
College degree or more	43.3%	45.3%	37.3%	47.2%		
Employed					1.19	0.553
No	66.3%	69.6%	64.6%	64.6%		
Yes	33.7%	30.4%	35.4%	35.4%		
Financial insecurity level	3.95 (±1.95)	4.24 (±2.09)	3.93 (±1.90)	3.68 (±1.83)	3.43	0.033
Residential rurality	3.11 (±1.43)	3.12 (±1.40)	3.19 (±1.46)	3.01 (±1.44)	0.61	0.544
Care recipient’s age	74.86 (±11.64)	76.65 (±12.00)	73.93 (±11.71)	74.00 (±11.04)	2.87	0.058
Cognitive status of care recipient					3.97	0.410
No Impairment	61.1%	55.9%	65.2%	62.1%		
Mild cognitive impairment	22.4%	24.8%	18.6%	23.6%		
Alzheimer’s disease or dementia	16.6%	19.3%	16.1%	14.3%		
Lives with care recipient					1.47	0.480
No	35.4%	31.7%	37.3%	37.3%		
Yes	64.6%	68.3%	62.7%	62.7%		
Hours of care given weekly	55.40 (±51.97)	59.62 (±55.20)	49.30 (±45.14)	57.28 (±54.69)	1.75	0.175
Social engagement activities	1.39 (±1.39)	1.24 (±1.30)	1.39 (±1.30)	1.54 (±1.53)	1.94	0.144
caregiving strain scale	3.28 (±2.49)	2.59 (±2.30)	3.60 (±2.50)	3.64 (±2.53)	9.49	<0.001
Satisfaction in caregiving support received	55.84 (±33.61)	53.08 (±36.48)	52.82 (±29.74)	61.63 (±33.71)	3.62	0.027

When comparing sample characteristics across the PTUC Scale tertiles, on average caregivers in the highest PTUC Scale tertiles were younger (*t* = 5.90, *p* = 0.003) and had lower financial insecurity levels (*t* = 3.43, *p* = 0.033). A significantly larger proportion of non-White participants were in the highest PTUC Scale tertile (χ^2^ = 30.39, *p* < 0.001). On average, caregivers in the highest PTUC Scale tertile had higher Caregiver Strain Scale scores (*f* = 9.49, *p* < 0.001) and higher satisfaction in the caregiving support they received (*f* = 3.62, *p* = 0.027).

Table 2 presents the ordinal regression model identifying factors associated with PTUC Scale scores among unpaid caregivers. Across tertiles, unpaid caregivers who were male (Beta = 0.422, *p* = 0.048), Hispanic (Beta = 0.779, *p* = 0.010), African American (Beta = 1.064, *p* < 0.001), and Asian or Pacific Islander (Beta = 0.958, *p* = 0.010) reported higher PTUC Scale scores. On average, lower PTUC Scale scores were reported among unpaid caregivers who were older (Beta = −0.018, *p* = 0.030) and those with higher financial insecurity levels (Beta = −0.010, *p* = 0.003). Unpaid caregivers with higher caregiver strain (Beta = 0.149, *p* < 0.001) and more satisfaction about support for caregiving from family/friends/neighbors (Beta = 0.009, *p* = 0.002) reported higher PTUC Scale scores. On average, lower PTUC Scale scores were reported among unpaid caregivers whose care recipients had more cognitive impairment (Beta = −0.245, *p* = 0.048).

**Table 2:** Ordinal regression: factors associated with higher PTUC Scale tertiles.

Variables				95% CI	
	B	S.E.	*P*	Lower	Upper
Age	−0.018	0.01	0.030	−0.04	0.00
Male	0.422	0.21	0.048	0.01	0.84
Hispanic	0.779	0.30	0.010	0.19	1.37
Race: White	–	–	–	–	–
Race: Black or African American	1.064	0.29	<0.001	0.51	1.62
Race: Asian or Pacific Islander	0.958	0.37	0.010	0.22	1.69
Race: other or multiple races	0.062	0.40	0.875	−0.71	0.84
Education: high school or less	–	–	–	–	–
Education: some college/associates	0.229	0.25	0.354	−0.26	0.72
Education: college degree or more	0.397	0.26	0.130	−0.12	0.91
Employed	−0.093	0.21	0.654	−0.50	0.31
Financial insecurity level	−0.161	0.05	0.003	−0.27	−0.05
Residential rurality	0.073	0.07	0.263	−0.05	0.20
Care recipient’s age	−0.010	0.01	0.256	−0.03	0.01
Cognitive status of care recipient	−0.245	0.12	0.048	−0.49	0.00
Lives with care recipient	−0.266	0.22	0.216	−0.69	0.16
Hours of care given weekly	0.000	0.00	0.803	0.00	0.00
Social engagement activities	0.054	0.07	0.425	−0.08	0.19
Caregiving strain scale	0.149	0.04	<0.001	0.07	0.23
Satisfaction in caregiving support received	0.009	0.00	0.002	0.00	0.01

Nagelkerke *R*^2^ = 0.181.

## 4 Discussion

The aim of this study was to identify the perceptions of unpaid caregivers on the usefulness of technology for caregiving and the factors that influence their perceptions. We found that unpaid caregivers who were younger and reported more satisfaction with the social support they receive had a higher perception of the usefulness of technology for caregiving. Similarly, unpaid caregivers who had higher caregiving strain also had a higher perception of the usefulness of technology in caregiving. Unpaid caregivers of individuals with more severe cognitive impairment, however, did not find technology for caregiving to be as useful.

In recent years, caregiving technologies have become increasingly prominent, accompanied by a heightened awareness about how their use varies across distinct populations and socioeconomic groups (3). Findings from a national caregiver survey highlighted notable disparities in reported technology usage (e.g., devices and specific functionalities) between caregivers and the individuals they support, as well as the factors linked to various patterns of technology adoption (22). Our study builds upon these findings by examining the perceived usefulness of technological solutions for caregiving, which has potential to influence uptake, duration of use, and overall care quality. Examples of technological innovations for caregiving encompass areas of diagnosis, evaluation and monitoring, functional support, recreational engagement, and overall care coordination (23). While the current study did not examine the perceived usefulness of technology for specific purposes, our study provides insights about the caregiving subgroups and caregiving contexts where such technologies may be more useful.

Age of the user plays a pertinent role in the acceptance of technological assistance in everyday life (24–26). Among unpaid caregivers, older age of the caregiver was associated with significantly lower perceptions of usefulness of caregiving technology. Studies which examined the acceptance and usefulness of caregiving technology among caregivers have consistently shown that younger caregivers are more accepting of caregiving technology (19, 27). Findings from our study suggest that younger unpaid caregivers may have a higher perception of technology’s usefulness for caregiving. The utility of technology to improve health outcomes is significantly associated with technology literacy and comfort in navigating digital tools (28). Prior research suggests that compared to younger individuals, older adults have lower technology literacy (25, 26), which could influence the perception of usefulness of these interventions for caregiving. Since the COVID-19 pandemic, older adults’ use of technology has increased and diversified (28, 29); yet, there still is persistent digital divide based on age. For example, 2021 Pew Research Center’s digital technology survey data showed 35 percentage point difference in the smartphone ownership between those at ages 18–29 years old and older adults at 65 years and older (30). In the current study, relative to their White counterparts, larger proportions of racial/ethnic minority caregivers (e.g., Hispanic, African American, and Asian or Pacific Islander) reported middle and highest PTUC Scale scores (relative to low PTUC Scale scores), respectively. These findings confirm those reported elsewhere (19). While the specific factors contributing to racial/ethnic differences in the perceived usefulness of technology for caregiving remain unclear (e.g., cultural attitudes, familial values), further research is warranted to explore and contextualize these differences.

In our study, perception of technology usefulness for caregiving was higher among individuals facing higher caregiving strain and those more satisfied with the help received from social networks to support their caregiving role. Similar findings have been reported in previous studies (22). Unpaid caregivers facing significant burden from caregiving are more likely to seek support to manage their caregiving roles (31), including being open to using technology to support caregiving. Caregivers reporting high burden and strain are also more likely to be engaged in providing long-term unpaid caregiving (32, 33) and may be in more complex care situations that may require additional skills in caregiving that they are often ill-prepared for (31). In these circumstances, technology to support caregiving may be perceived to be highly useful in supporting their caregiving roles.

Caregivers with strong support networks are more likely to have support to address issues that might limit access to, and utility of, technology to support caregiving. Adoption of technology and its use to support caregiving has been shown to be reliant of the caregivers’ external sources of information about available technology (34), indicating the role of social networks in increasing uptake of technology for caregiving. Moreover, utility of technology for caregiving is also dependent on the user’s technology literacy (16, 35, 36), access to broadband internet, and the affordability of the technology in question (26). Caregivers with robust support networks may be able to navigate these potential barriers more easily.

Additionally, in our study, unpaid caregivers of individuals with cognitive impairment were less likely to perceive technology for caregiving as useful. Studies have identified numerous factors that influence the adoption of technology among caregivers of individuals with cognitive impairment (37–39). For example, the timing and pace of technology introduction, as well as disease progression, are important considerations that may impact the user’s level of comfort, familiarity, and ability to adapt to devices (36). Among caregivers of individuals with cognitive impairment, the limitation in their participation in utilizing certain technologies for caregiving may influence the reach and effectiveness of the caregiving technology and often lead to missed opportunities in using technology to support caregiving. Typically, caregivers of individuals with cognitive impairment are most receptive when technology is introduced at earlier stages of disease progression (37).

Digital literacy and technology anxiety also contribute to the user’s ability to adapt to the technology (38). Caregiving technology may require an initial investment in terms of time and effort to establish and learn the technology. Furthermore, for caregivers caring for those with cognitive decline, the need to communicate about or implement a new technology in a daily routine can be perceived as a challenge, resulting in the lower perception of the usefulness of technology for caregiving. To ensure successful adoption, assistive devices should align with the needs, preferences, and abilities of the user and be capable of adapting to dementia in its different stages (36). Studies have also shown that among caregivers of people with dementia, the perception of the usefulness of technology for caregiving also depends on the type of technology (38). These finding underscore the importance of adequately considering the needs and preferences of the caregiver and individual with cognitive impairment to enhance product utility and use (39).

### 4.1 Limitations

This study has limitations, which should be acknowledged. Data were cross-sectional; therefore, associations could be identified but causality could not be inferred. Data were collected using an internet-delivered survey from a Qualtrics panel sample. While the quota sampling strategy yielded a large and somewhat diverse national sample of unpaid caregivers, this sample may not be representative of all unpaid caregivers in the United States. Recruiting participants through Qualtrics may have introduced self-selection bias based on topical interest and may have included caregivers with higher digital literacy than the general caregiving population. Although the perceived usefulness of technology for caregiving was identified using the PTUC scale, this study did not directly assess whether the unpaid caregivers were using various forms of technology for specific caregiving purposes. Research does suggest, however, that perceived usefulness of technology contributes to its adoption (10), which may indicate that some participants in the current study were already using technology for caregiving. Perceived usefulness and adoption of technology is impacted by individual level (e.g., cost, time, and adaptability to change) and structural-level factors (e.g., broadband accessibility, technology support, and workplace policies) that may hinder adoption (3). Because perceived usefulness of technology may not always translate into its actual adoption or utilization, future studies should examine the concordance between these two factors while carefully considering contextual influences. This study asked caregivers to report about their care recipients’ personal characteristics and their current caregiving situation. However, additional potentially important information may not have been collected, which could better contextualize the perceived usefulness of caregiving technology (e.g., their care recipients’ health conditions, diagnoses, impairments, or behavioral risks [e.g., wandering]; whether the caregiver shares caregiving responsibilities with others). The current sample primarily contained caregivers who resided in urban areas. Given caregivers in rural areas may be more susceptible to the digital divide (e.g., less affluence and unreliable internet) and may have unique caregiving-related responsibilities based on geospatial circumstances (e.g., dispersion of health resources, size of residential properties, roles pertaining to property upkeep), rural–urban differences in perceived technology usefulness were anticipated but not observed. Future research should examine the usefulness of technologies from the perspective of caregivers in rural versus urban areas using more geographically diverse samples.

## 5 Conclusion

This cross-sectional study examined the perceptions of unpaid caregivers on the usefulness of technology for caregiving and the factors that predicted their perceptions. Caregivers reported about the usefulness of technology for caregiving to improve their relationship and communication with the care recipient, help their care recipients live more independently, and ease the burden of caregiving. Experiencing greater caregiver strain, greater satisfaction with the social support received, and lower financial insecurity were associated with higher perceptions of the usefulness of technology for caregiving among unpaid caregivers in terms of needs and perceived benefits. However, caregivers caring for individuals with more severe cognitive decline did not find technology for caregiving to be as useful. This study demonstrates the importance of having caregivers and those being cared for participate more deeply in the design and evaluation of the utility of assistive technologies. By identifying predictors of the usefulness of technology, more targeted and tailored technology interventions can be designed and adopted by diverse unpaid caregivers; thus, improving their quality of life.

## Data Availability

The raw data supporting the conclusions of this article will be made available by the authors, without undue reservation.

## Footnotes

^1^ https://www.ibm.com/products/spss-statistics

## References

[1] AARP, National Alliance for caregiving: caregiving in the United States 2020. (2020). Available online at: www.greenwaldresearch.com (Accessed February 14, 2025).

[2] Freedman VA Patterson SE Cornman JC Wolff JL. A day in the life of caregivers to older adults with and without dementia: comparisons of care time and emotional health. Alzheimers Dement. (2022) 18:1650–61. doi: 10.1002/alz.12550, PMID: 35103394 PMC9339593

[3] Lindeman DA Kim KK Gladstone C Apesoa-Varano EC. Technology and caregiving: emerging interventions and directions for research. Gerontologist. (2020) 60:S41–9. doi: 10.1093/geront/gnz178, PMID: 32057082 PMC7019659

[4] Del-Pino-Casado R Priego-Cubero E López-Martínez C Orgeta V. Subjective caregiver burden and anxiety in informal caregivers: a systematic review and meta-analysis. PLoS One. (2021) 16:e0247143. doi: 10.1371/journal.pone.0247143 33647035 PMC7920375

[5] Gilhooly KJ Gilhooly MLM Sullivan MP McIntyre A Wilson L Harding E. A meta-review of stress, coping and interventions in dementia and dementia caregiving. BMC Geriatr. (2016) 16:106. doi: 10.1186/s12877-016-0280-8, PMID: 27193287 PMC4872341

[6] Koyama A Matsushita M Hashimoto M Fujise N Ishikawa T Tanaka H. Mental health among younger and older caregivers of dementia patients. Psychogeriatrics. (2017) 17:108–14. doi: 10.1111/psyg.12200, PMID: 26968528

[7] Sörensen S Duberstein P Gill D Pinquart M. Dementia care: mental health effects, intervention strategies, and clinical implications. Lancet Neurol. (2006) 5:961–73. doi: 10.1016/S1474-4422(06)70599-3, PMID: 17052663

[8] Lwi SJ Ford BQ Casey JJ Miller BL Levenson RW. Poor caregiver mental health predicts mortality of patients with neurodegenerative disease. Proc Natl Acad Sci USA. (2017) 114:7319–24. doi: 10.1073/pnas.1701597114, PMID: 28655841 PMC5514722

[9] Yang P Bi G Qi J Wang X Yang Y Xu L. Multimodal wearable intelligence for dementia Care in Healthcare 4.0: a survey. Inf Syst Front. (2021) 27:197–214. doi: 10.1007/s10796-021-10163-3

[10] Sriram V Jenkinson C Peters M. Informal carers’ experience of assistive technology use in dementia care at home: a systematic review. BMC Geriatr. (2019) 19:160. doi: 10.1186/s12877-019-1169-0, PMID: 31196003 PMC6567448

[11] Madara MK. Assistive technologies in reducing caregiver burden among informal caregivers of older adults: a systematic review. Disabil Rehabil Assist Technol. (2016) 11:353–60. doi: 10.3109/17483107.2015.1087061, PMID: 26371519

[12] Ben MW Pysklywec A Fuhrer MJ Jutai JW Plante M Demers L. Caregivers’ experiences with the selection and use of assistive technology. Disabil Rehabil Assist Technol. (2018) 13:562–7. doi: 10.1080/17483107.2017.1353652, PMID: 28768438

[13] Choukou MA Olatoye F Urbanowski R Caon M Monnin C. Digital health technology to support health care professionals and family caregivers caring for patients with cognitive impairment: scoping review. JMIR Ment Health. (2023) 10:e40330. doi: 10.2196/40330, PMID: 36630174 PMC9878361

[14] Gitlin LN Bouranis N Kern V Koeuth S Marx KA McClure LA. WeCareAdvisor, an online platform to help family caregivers manage dementia-related behavioral symptoms: an efficacy trial in the time of COVID-19. J Technol Behav Sci. (2022) 7:33–44. doi: 10.1007/s41347-021-00204-8 33786370 PMC7994055

[15] Kales HC Gitlin LN Stanislawski B Myra Kim H Marx K Turnwald M. Effect of the WeCareAdvisor™ on family caregiver outcomes in dementia: a pilot randomized controlled trial. BMC Geriatr. (2018) 18:113. doi: 10.1186/s12877-018-0801-8, PMID: 29747583 PMC5946471

[16] Albers EA Mikal J Millenbah A Finlay J Jutkowitz E Mitchell L. The use of technology among persons with memory concerns and their caregivers in the United States during the COVID-19 pandemic: qualitative study. JMIR Aging. (2022) 5:e31552. doi: 10.2196/31552, PMID: 35134748 PMC8972107

[17] Chirico I Giebel C Lion K Mackowiak M Chattat R Cations M. Use of technology by people with dementia and informal carers during COVID-19: a cross-country comparison. Int J Geriatr Psychiatry. (2022) 37:1–10. doi: 10.1002/gps.5801, PMID: 36005276

[18] Lee S Ory MG Dahlke DV Smith ML. Social support, sense of belonging, and communication technology use among paid and unpaid caregivers of middle-aged and older adults. Front. Public Health. (2022) 202:10–1. doi: 10.1016/j.puhe.2021.01.009, PMID: 35712314 PMC9196896

[19] Vollmer Dahlke D Lee S Smith ML Shubert T Popovich S Ory MG. Attitudes toward technology and use of fall alert wearables in caregiving: survey study. JMIR Aging. (2021) 4:e23381. doi: 10.2196/23381, PMID: 33502320 PMC8081189

[20] The Atlantic Philanthropies NCOA issues call-to-action for National Chronic Care Reform Based on survey of Americans with chronic conditions. (2009) Available online at: https://www.atlanticphilanthropies.org/news/video-ncoa-issues-call-action-national-chronic-care-reform-based-results-comprehensive-survey-a (Accessed February 16, 2025).

[21] Meek K Bergeron C Towne S Ahn S Ory M Smith M. Restricted social engagement among adults living with chronic conditions. Int J Environ Res Public Health. (2018) 15:158. doi: 10.3390/ijerph15010158, PMID: 29351193 PMC5800257

[22] Lee S Smith M Dahlke DV Shubert T Popovich S Ory M. Technology use among caregivers for persons with and without cognitive impairment. Innov Aging. (2020) 4:468–9. doi: 10.1093/geroni/igaa057.1517, PMID: 38779614

[23] Astell AJ Bouranis N Hoey J Lindauer A Mihailidis A Nugent C. Technology and dementia professional interest area … technology and dementia: the future is now, Technology and dementia: the future is now. Dement Geriatr Cogn Disord. (2019) 47:131–9. doi: 10.1159/000497800, PMID: PMC6643496 31247624

[24] Claes V Devriendt E Tournoy J Milisen K. Attitudes and perceptions of adults of 60 years and older towards in-home monitoring of the activities of daily living with contactless sensors: an explorative study. Int J Nurs Stud. (2015) 52:134–48. doi: 10.1016/j.ijnurstu.2014.05.010 24951084

[25] Arcury TA Sandberg JC Melius KP Quandt SA Leng X Latulipe C. Older adult internet use and eHealth literacy. J Appl Gerontol. (2020) 39:141–50. doi: 10.1177/0733464818807468, PMID: 30353776 PMC6698430

[26] Fields J Cemballi AG Michalec C Uchida D Griffiths K Cardes H. In-home technology training among socially isolated older adults: findings from the tech allies program. J Appl Gerontol. (2021) 40:489–99. doi: 10.1177/0733464820910028, PMID: 32141373

[27] Schulz R Beach SR Matthews JT Courtney K De Vito DA Mecca LP. Caregivers’ willingness to pay for technologies to support caregiving. Gerontologist. (2016) 56:817–29. doi: 10.1093/geront/gnv033, PMID: 26035899 PMC5019044

[28] Guan C Bouzida A Oncy-Avila RM. Taking an (embodied) cue from community health: designing dementia caregiver support technology to advance health equity In:. Conference on Human Factors in Computing Systems - Proceedings. New York, NY, USA: Association for Computing Machinery (2021). 1–16.

[29] Kakulla B. 2022 tech trends and adults 50-plus. Washington, DC: AARP Research. (2021). doi: 10.26419/res.00493.001

[30] Faverio M. Share of those 65 and older who are tech users has grown in the past decade (2022). Available online at: https://www.pewresearch.org/short-reads/2022/01/13/share-of-those-65-and-older-who-are-tech-users-has-grown-in-the-past-decade/ (Accessed February 14, 2025).

[31] Reinhard SC Caldera S Houser A Choula RB. Valuing the invaluable: 2023 update strengthening supports for family caregivers. AARP Public Policy Institute, 10. (2023). Available at: https://www.archrespite.org/wp-content/uploads/2023/03/valuing-the-invaluable-2023-update.doi_.10.26419-2Fppi.00082.006.pdf

[32] Park C Shin DW Choi JY Kang J Baek YJ Mo HN. Determinants of the burden and positivity of family caregivers of terminally ill cancer patients in Korea. Psychooncology. (2012) 21:282–90. doi: 10.1002/pon.1893 22383270

[33] Park SS. Caregivers’ mental health and somatic symptoms during COVID-19. J Gerontol B. (2021) 76:e235–40. doi: 10.1093/geronb/gbaa121 PMC7454918 32738144

[34] Xiong C D’Souza A El-Khechen-Richandi G Mihailidis A Cameron JI Astell A. Perceptions of digital technology experiences and development among family caregivers and technology researchers: qualitative study. JMIR Form Res. (2022) 6:e19967. doi: 10.2196/19967, PMID: 35089150 PMC8838597

[35] Finkelstein R Wu Y Brennan-Ing M. Older adults’ experiences with using information and communication technology and tech support services in New York City: findings and recommendations for post-pandemic digital pedagogy for older adults. Front Psychol. (2023) 14:1129512. doi: 10.3389/fpsyg.2023.1129512, PMID: 37138998 PMC10150999

[36] Bastoni S Wrede C da Silva MC Sanderman R Gaggioli A Braakman-Jansen A. Factors influencing implementation of eHealth technologies to support informal dementia care: umbrella review. JMIR Aging. (2021) 4:e30841. doi: 10.2196/30841, PMID: 34623314 PMC8538023

[37] Boyle LD Husebo BS Vislapuu M. Promotors and barriers to the implementation and adoption of assistive technology and telecare for people with dementia and their caregivers: a systematic review of the literature. BMC Health Serv Res. (2022) 22:1573. doi: 10.1186/s12913-022-08968-2, PMID: 36550456 PMC9780101

[38] Cahill S Begley E Faulkner JP Hagen I. “It gives me a sense of independence” – findings from Ireland on the use and usefulness of assistive technology for people with dementia. Technol Disabil. (2007) 19:133–42. doi: 10.3233/TAD-2007-192-310, PMID: 39743787

[39] Suijkerbuijk S Nap HH Cornelisse L Ijsselsteijn WA YAW DK MMN M. Active involvement of people with dementia: a systematic review of studies developing supportive technologies. J Alzheimers Dis. (2019) 69:1041–65. doi: 10.3233/JAD-190050, PMID: 31156158 PMC6597993

